# NGS for the diagnosis of autoinflammatory diseases: the experience of Montpellier

**DOI:** 10.1186/1546-0096-13-S1-O17

**Published:** 2015-09-28

**Authors:** G Sarrabay, G Tachon, D Mechin, I Touitou

**Affiliations:** 1 CHRU Montpellier, Montpellier, France

## Introduction

Monogenic autoinflammatory diseases present with overlapping clinical features such as recurrent fever, biological inflammation, abdominal pain, arthritis, and sometimes disabling complications. Over 30 genes have been confirmed or hypothesized as causing diseases. Sanger sequencing is the gold-standard approach for genetic diagnosis, but cannot be exhaustive.

## Objectives

We aimed at offering a quick and efficient service for these genetically heterogeneous disorders through NGS sequencing of all published and candidate (N=32) autoinflammatory genes.

## Patients and methods

34 patients were selected on clinical grounds by members of the French reference center for autoinflammatory diseases (CeReMAI). 9 of them were considered positive control samples as they had previously known variants (by Sanger sequencing) and were run in parallel for methodological validation.

A custom panel of 108kb was designed to capture the genomic regions of interest using the Illumina Nextera Rapid Capture enrichment DNA preparation kit. MicroV2 chip were then loaded with 12 samples on a MiSeq equipment for multiparallel sequencing. Annotation and filtering were performed with Seqnext software (JSI), and annotation was performed with Alamut Visual (Interactive Biosoftware).

## Results

We obtained a 545X average coverage and only 2 exons were not captured. Minimal coverage was 40X. Results were concordant in 100% of the cases with Sanger analysis. We uncovered pathological mutations or variants of unknown significance in 17 patients. Of note recurrent genes were *CARD14, NOD2, PSTPIP1* and *SCL29A3*. About 20 new potentially pathogenic sequence variants were found in published genes.

## Conclusion

This innovative sequencing approach demonstrated high performance (accuracy, the precision, analytical sensitivity and specificity) in evaluating mutations in known autoinflammatory genes. The current positive rate using sequential Sanger sequencing of 1-4 genes in the French network is quite stable around 10-15%. The NGS approach allowed diagnosis of approximately another 20% of patients.

